# Multipotent/pluripotent stem cell populations in stromal tissues and peripheral blood: exploring diversity, potential, and therapeutic applications

**DOI:** 10.1186/s13287-024-03752-x

**Published:** 2024-05-12

**Authors:** Domenico Aprile, Deanira Patrone, Gianfranco Peluso, Umberto Galderisi

**Affiliations:** 1 Department of Experimental Medicine, Luigi Vanvitelli Campania University, Naples, Italy; 2 Faculty of Medicine and Surgery, Saint Camillus International, University of Health Sciences, Rome, Italy; 3 Genome and Stem Cell Center (GENKÖK), Erciyes University, Kayseri, Turkey; 4 Center for Biotechnology, Sbarro Institute for Cancer Research and Molecular Medicine Temple University, Philadelphia, PA USA

**Keywords:** Stem cells, MUSE cells, VSEL, SBSC, MIAMI cells, MAPC, Pluripotency

## Abstract

The concept of “stemness” incorporates the molecular mechanisms that regulate the unlimited self-regenerative potential typical of undifferentiated primitive cells. These cells possess the unique ability to navigate the cell cycle, transitioning in and out of the quiescent G0 phase, and hold the capacity to generate diverse cell phenotypes. Stem cells, as undifferentiated precursors endow with extraordinary regenerative capabilities, exhibit a heterogeneous and tissue-specific distribution throughout the human body. The identification and characterization of distinct stem cell populations across various tissues have revolutionized our understanding of tissue homeostasis and regeneration. From the hematopoietic to the nervous and musculoskeletal systems, the presence of tissue-specific stem cells underlines the complex adaptability of multicellular organisms. Recent investigations have revealed a diverse cohort of non-hematopoietic stem cells (non-HSC), primarily within bone marrow and other stromal tissue, alongside established hematopoietic stem cells (HSC). Among these non-HSC, a rare subset exhibits pluripotent characteristics. In vitro and in vivo studies have demonstrated the remarkable differentiation potential of these putative stem cells, known by various names including multipotent adult progenitor cells (MAPC), marrow-isolated adult multilineage inducible cells (MIAMI), small blood stem cells (SBSC), very small embryonic-like stem cells (VSELs), and multilineage differentiating stress enduring cells (MUSE). The diverse nomenclatures assigned to these primitive stem cell populations may arise from different origins or varied experimental methodologies. This review aims to present a comprehensive comparison of various subpopulations of multipotent/pluripotent stem cells derived from stromal tissues. By analysing isolation techniques and surface marker expression associated with these populations, we aim to delineate the similarities and distinctions among stromal tissue-derived stem cells. Understanding the nuances of these tissue-specific stem cells is critical for unlocking their therapeutic potential and advancing regenerative medicine. The future of stem cells research should prioritize the standardization of methodologies and collaborative investigations in shared laboratory environments. This approach could mitigate variability in research outcomes and foster scientific partnerships to fully exploit the therapeutic potential of pluripotent stem cells.

## Background

The term “stemness” refers to the molecular unlimited self-regenerative process, triggered by undifferentiated primitive cells that have the ability to enter and exit the G0 phase of the cell cycle and the power to generate one or more cell phenotypes. Stem cells, undifferentiated progenitors endowed with remarkable regenerative potential, display a diverse and tissue-specific distribution throughout the human body. The identification and characterization of distinct stem cell populations across various tissues have transformed our comprehension of tissue homeostasis and regeneration [1]. From the hematopoietic system to the nervous and musculoskeletal systems, the presence of tissue-specific stem cells underscores the complexity and adaptability of multicellular organisms. Notably, accumulating evidence emphasizes that different tissues harbor unique types of stem cells, each equipped with specialized functions tailored to the specific microenvironment of their residing niche. Understanding the intricate traits of tissue-specific stem cells is vital for propelling regenerative medicine forward and unlocking their full therapeutic capabilities [2].

Stem cells are characterized by their unique ability to undergo continuous division (self-renewal) and, upon specific cues, differentiate into various specialized cell types [3]. Classification of stem cells is based on their differentiation potential, encompassing totipotent and pluripotent stem cells associated with early embryo development, as well as multipotent, oligopotent, and unipotent stem cells prevalent in adult tissues, each displaying varying degrees of differentiation capacity. Totipotent cells are present in the earliest developmental stages, whereas pluripotent cells are located in the inner cell mass of the blastocyst, capable of giving rise to a diverse array of cell types representing the three germ layers but not extraembryonic tissues. Multipotent stem cells, such as mesenchymal and hematopoietic stem cells, can differentiate into various cell types within the same embryonic germ layer. Oligopotent and unipotent stem cells, often referred to as progenitor cells, have limited differentiation potential [4].

The classification of stem cells based on their potency often arises from in vitro experiments demonstrating their differentiation potential. However, for some stem cells, the in vitro differentiation potential may not accurately reflect their in vivo differentiation capacities. Mesenchymal stromal cells (MSCs) encompass a stem cell population capable of in vivo differentiation into mesodermal derivatives, such as osteocytes, adipocytes, and chondrocytes. Nevertheless, certain in vitro studies have indicated that these cells may also differentiate into neurons and other ectodermal and/or endodermal cell phenotypes.

Discrepancies between in vitro and in vivo physiological differentiation potential, along with variations in in vitro properties depending on isolation and cultivation procedures, may lead to the misclassification of stem cells [5]. In this scenario, it is important to emphasize that the in vitro biological properties of stem cells may not necessarily reflect their physiological functions in the body. The classification of stem cells for investigations aimed at isolating and cultivating stem cells for therapeutic purposes must not be confused with studies devoted to dissecting the physiological role of stem cells in tissue renewal and organismal homeostasis.

Recent discoveries suggest the existence of a diverse group of non-hematopoietic stem cells (non-HSC) primarily within bone marrow stromal tissue, as well as in the stromal components of other tissues, alongside well-established hematopoietic stem cells (HSC). Moreover, it has been theorized that among these non-HSC, a particularly rare subset exhibits several characteristics of pluripotent stem cells (PSC). In vitro studies have demonstrated the capacity of these potential PSC to differentiate into cells representing all three germ layers, and they have been identified in the scientific literature under various names, including i) multipotent adult progenitor cells (MAPC), ii) marrow-isolated adult multilineage inducible (MIAMI) cells, iii) small blood stem cell (SBSC), iv) very small embryonic-like stem cells (VSELs), and v) Multilineage differentiating stress enduring (MUSE) cells. The assignment of different nomenclatures to similar or overlapping populations of primitive stem cells within the BM may stem from diverse experimental approaches [3–6].

## Aim

This review aims to provide a comprehensive report and comparison of various subpopulations of multipotent/pluripotent stem cells derived from stromal tissues. Through an analysis of the isolation methods and surface marker expression associated with these diverse populations, the goal is to develop a thorough understanding of the commonalities and differences among stem cells found in stromal tissues. This is to ascertain whether stem cells isolated using different methods and subsequently labeled with distinct names may refer to a singular core population of stem cells. In this scenario, the diverse biological properties of the various non-HSC stem cells may depend on the percentage of stem cells and progenitors present in the samples isolated using different procedures. It should be noted that even stem cells isolated at the 'purest' grade contain several subpopulations, including both stem cells and lineage-committed cells at various degrees of maturation [7, 8].

## Stromal cell population

The term “stromal cells” encompasses a diverse group of connective tissue cells forming the structural framework for organs and playing crucial roles in health and disease. Coexisting with parenchymal cells that define organ-specific functions, stromal cell populations include fibroblasts, pericytes, and telocytes found across various organ systems, alongside with heterogenous cell populations such as bone-marrow-derived mesenchymal stem/stromal cells (MSCs) and adipose tissue-derived stem/stromal cells (ASCs), which contain stem cells, progenitor cells and other stromal cell types [9–11]. Recent research has unveiled the molecular underpinnings of stromal cell contributions to processes such as tissue development, homeostasis, regeneration, immune responses, cancer, and disease [12]. While stromal cells shape their microenvironment, their functions are profoundly influenced by tissue context.

The intricate landscape of stromal cells unveils a diverse array of subpopulations, including MUSE cells, MIAMI cells, MAPCs and VSELs, each holding unique characteristics within the realm of adult multipotent/pluripotent stem cells. These distinct entities, nestled within the stromal tissues, add a layer of complexity to our understanding of the physiological regenerative potential of our tissues and organs [13]. The recognition and exploration of these specific multipotent/pluripotent subpopulations within stromal tissues broaden the horizons of regenerative medicine, offering promising avenues for therapeutic applications. Harnessing the regenerative capabilities of MUSE cells, MIAMI cells, MAPCs and VSELs, from stromal environments could pave the way for innovative approaches in tissue repair and disease treatment, elevating the role of stromal cells as crucial players in the pursuit of regenerative medicine breakthroughs.

### Multilineage-differentiating stress enduring (MUSE) cells

In 2010, Professor Dezawa's research group at the University of Sendai, Japan, successfully isolated a distinctive stem cell population termed Multilineage-differentiating Stress Enduring cells (MUSE) from the mononuclear cell fraction of the bone marrow [14]. These endogenous, stress-resistant stem cells express pluripotency master genes and feature specific surface markers like SSEA-3 [15, 16]. The utilization of FACS and MACS in isolation protocols ensures the dependable purification of MUSE cells.

MUSE cells were initially identified in humans and mice, and subsequently in other species, including rat, rabbit, sheep, and monkey, demonstrating evolutionary conservation and broad applicability [17, 18]. These cells are found in various tissues, such as fibroblasts, adipose tissue and bone marrow MSCs, and peripheral blood, showcasing their versatile accessibility for therapeutic purposes [19].

In vitro, MUSE cells exhibit extensive trilineage differentiation into hepatocytes, neural/neuronal-lineage cells, cardiomyocytes, skeletal muscle, and glomerular cells, highlighting their pluripotent nature. Their distinctive ability for clonal in vitro trilineage differentiation, as confirmed by RT-qPCR and ICC analyses, underscores their unique pluripotent characteristics, crucial for controlled and specific differentiation in therapeutic applications. In vivo, MUSE cells differentiate into diverse cell types, emphasizing their potential in regenerative medicine.

**Fig. 1:**
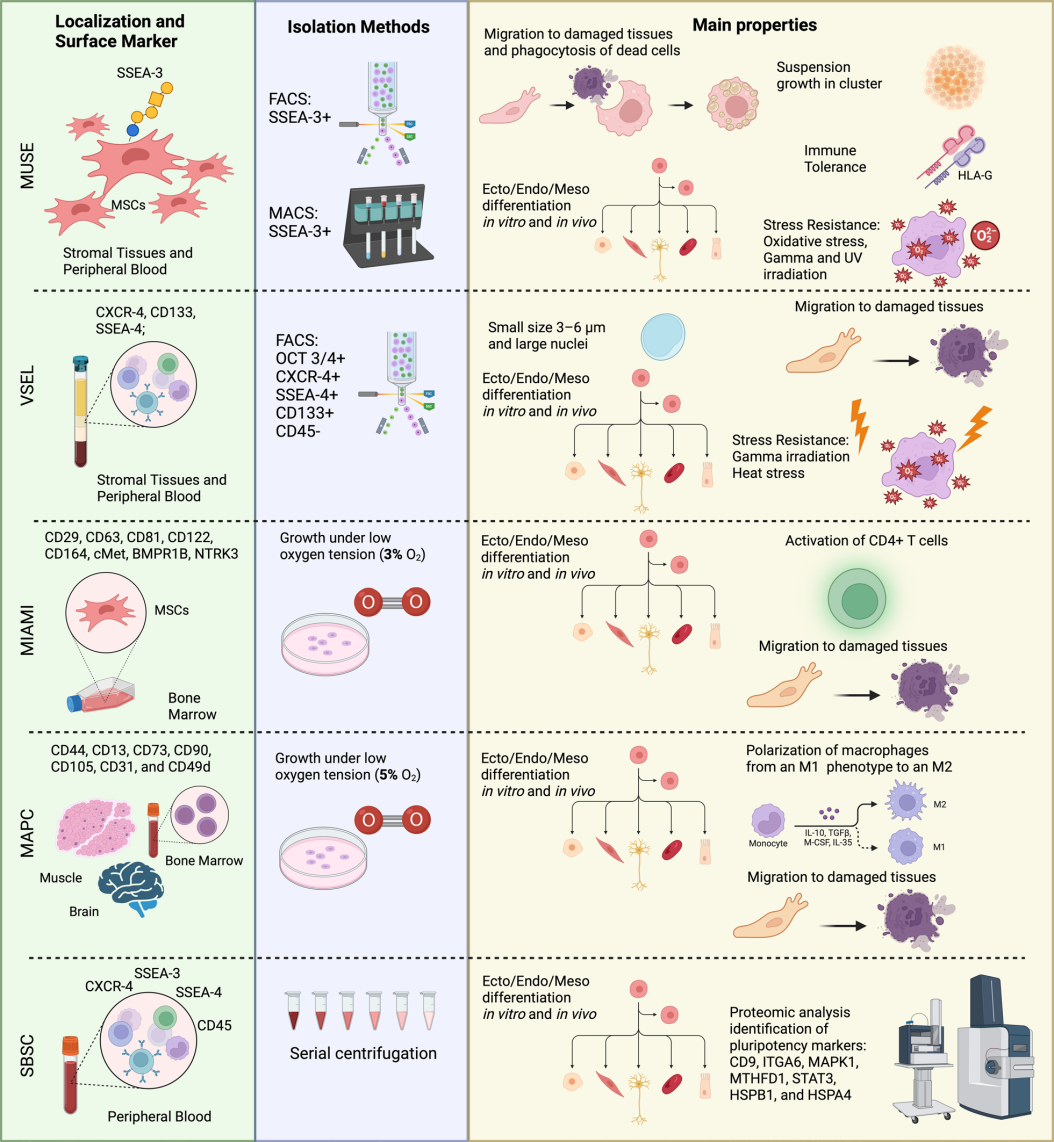
Key Properties of Stromal and Peripheral Blood Stem Cell Populations (MUSE, VSEL, SSBC, MIAMI, MAPC). Localization, surface markers, isolation methods, and main properties of stem cells have been reported

MUSE cells, present in connective tissues, remain quiescent but activate under stress conditions [20]. Their homing ability to damaged tissues and resistance to stressors like H_2_O_2_ and UV make them advantageous for therapeutic applications [21]. MUSE cells grow as single cells in suspension and form clusters positive for both MSC marker CD105 and SSEA-3. Upon transfer to an adhesion system, single cells express markers of mesoderm, ectoderm, and endoderm, indicating trilinear differentiation and self-renewal ability through multiple culture cycles (Table 1, Fig. 1).

**Table 1:** Main properties, isolation method, pheno/genotypic and functional characteristics of MUSE cells, VSEL, SBSC, MIAMI cells, MAPC

Cell type	Species	Localization	Isolation protocol	Surface markers	Other markers	Homing following damage	Stress resistance
MUSE cells	Human [14] Mouse [17] Rat, Rabbit, Sheep, Monkey [68]	Fibroblast [18] MSCs [14] Adipose Tissue [69] Peripheral Blood [19]	FACS [18] MACS [16]	SSEA-3 [14] CD105 [18, 31]	*OCT ¾, SOX-2, NANOG* [14, 16]	Migration to damage tissues [21]	After H_2_O_2_ and UV treatment [20]
VSEL	Mouse [32] Human [35]	Bone Marrow [32, 33] Umbilical cord blood [35] Peripheral blood [34]	FACS [32, 35] Ficoll-gradient centrifugation [34]	SSEA-1 [32] CXCR4 [32] CD133 [35] SSEA-4 [32, 34]	*OCT-4* [32]	Migration assay to ‘matrigel drop’ [32] Migration to damage tissues [42]	After high dose of gamma-irradiation (1500 cGy) [40] Resistant to extrinsic heat stress [70]
MIAMI cells	Human [48]	Bone Marrow [50]	Bone marrow isolation under low oxygen tension (3% O2) [48]	CD122, CD29, CD63, CD81, CD164, CD90, SSEA-4 [46, 48]	OCT3/4, NANOG, C-MET, REX1, SOX2, BMPR1B, POU4F1 [48]	Migration to damage tissues [50, 52]	Not reported
MAPC	Human [71] Mouse, Rat [56]	Bone Marrow, Brain, Muscle tissue [53] Bones [72]	Bone marrow isolation under 5% oxygen [56]	CD44, CD13, CD73, CD90, CD105, CD31, CD49d [54, 71, 72]	*FLK1 DIM, C-KIT, OCT4* [55]	Migration to damage tissues [73, 74]	Not reported
SBSC	Human [44]	Peripheral blood [44, 46]	Serial Centrifugation [44, 46]	SSEA-3, SSEA-4, CXCR4, CD 105 [44]	*NANOG, OCT4, SOX2, KLF4* [44]	Not reported	Not reported

Cell type	Bulk in vitro ecto/endo/meso differentiation	Clonal in vitro ecto/endo/meso differentiation	Bulk in vivo differentiation	Proteome analysis	Transcriptome analysis	Secretome analysis	Immunomodulatory capacity
MUSE cells	Demonstrated by RT-qPCR, WB and ICC [31]	Demonstrated by RT-qPCR and ICC [14, 16]	Hepatocyte [75] Neural/ neuronal-lineage cells [25, 29] Cardiomyocyte [28] Skeletal muscle [14] Glomerular cells [29]	Yes [22]	Yes [66]	Yes [20]	Activate regulatory T cells and suppress dendritic cell differentiation [21] Expression of human leukocyte antigen (HLA)-G [29]
VSEL	Demonstrated by RT-qPCR and ICC [32]	Not reported	Bone-like structures [76] Endotelial cells [77] Cardiomyocytes [40] Hepatocyte [78] Pancreatic cells [42]	Yes [38]	Yes [36, 79]	Not reported	Non-immunogenic [39]
MIAMI cells	Demonstrated by RT-qPCR and ICC [47]	Not reported	Osteoblastic and Neural cells [47] Endothelial cells [52] Neural cells [49]	Yes [80]	Not reported	Yes[51, 80]	Activation of CD4 + T cells [51]
MAPC	Demonstrated by RT-qPCR [55] WB and ICC [81]	Not reported	Endothelial cells [57] Linfo-hematopoietic cells [82]	Not reported	Yes [56]	Yes [58, 71]	Non-immunogenic inhibiting T cell proliferation Polarization of macrophages from an M1 phenotype to an M2 [58]
SBSC	Demonstrated by RT-qPCR and ICC [44]	Not reported	Osseointegration in dental implantation [46]	Yes [44]	Not reported	Not reported	Not reported

Comprehensive analyses of MUSE cells have included investigations into their transcriptomics and proteomics, identifying specific factors within the secretome that significantly contribute to stress resistance and tissue repair mechanisms [22]. The secretome of MUSE cells reveals a unique protein profile, including Serpins and 14-3-3 proteins, which contribute to stress tolerance and apoptosis inhibition [23]. Notably, in severe tissue damage, MUSE cells migrate from the bone marrow to peripheral blood, guided by the sphingosine-1-phosphate (S1P) pathway, particularly the S1PR2 receptor. In injured areas, MUSE cells persist and phagocyte apoptotic differentiated cells using distinct phagocytic receptor subsets compared to macrophages. The phagocyted contents from the differentiated cells, such as transcription factors, were promptly discharged into the cytoplasm, moved into the nucleus, and attached to the promoter regions of the stem cell genomes. Within 2 days, the MUSE cells exhibited lineage-specific markers associated with the phagocyted differentiated cells [21, 24]. MUSE cells exhibit immunomodulatory capacity, activating regulatory T cells, suppressing dendritic cell differentiation, and expressing HLA-G, suggesting potential in immune tolerance [25].

MUSE cells possess significant regenerative potential, demonstrated by their safety, accessibility, in vivo differentiation, and therapeutic effects in various models, including lacunar infarction, acute myocardial infarction, amyotrophic lateral sclerosis, and liver fibrosis [26–31]. Clinical trials for conditions like acute myocardial infarction, ischemic stroke, epidermolysis bullosa, spinal cord injuries, ALS, cerebral palsy, and COVID-19-related acute respiratory distress syndrome are underway in Japan. The absence of tumorigenesis post-injection and their applicability without gene manipulation or cytokine treatment further underscore MUSE cells' promise in regenerative medicine.

In summary, MUSE cells present a unique pluripotent profile, making them versatile candidates for regenerative medicine with applications in diverse biological contexts.

### Very small embryonic-like (VSEL) stem cells

Discovered in 2006, Very Small Embryonic-Like (VSEL) stem cells have emerged as a unique subset within the realm of stem cell research, holding great promise for regenerative medicine [32]. Unlike conventional stem cell populations, VSEL cells present distinct characteristics that set them apart and offer an ethical alternative to embryonic stem cells. VSEL cells, remarkably small in size (3–6 μm), lack certain lineage markers (CD45–, lin–), and express specific surface antigens like Sca-1 and CXCR4. They also exhibit pluripotency markers, including Oct-4, SSEA-3, Nanog, and Rex1, commonly associated with embryonic stem cells [33]. VSELs have been identified in both mice and humans, displaying remarkable differentiation potential and widespread localization in tissues such as bone marrow, umbilical cord blood, and peripheral blood. The sorting method involves the use of beads of predefined sizes, isolating Oct-4 + CXCR4 + SSEA-1 + Sca-1 + CD45 – lin – cells in mice and Oct-4 + CXCR4 + SSEA-4 + CD133 + CD45 – lin – cells in humans [34, 35]. These sorted cells display large nuclei with unorganized chromatin (euchroma). Approximately 5–10% of isolated VSELs, when cultured, can form VSEL-Derived Sphere (DS), resembling embryoid bodies. These structures, observed in young mice, exhibit features such as immature cells with large nuclei, and express pluripotency markers. VSEL-DS, when replated, can generate new secondary or tertiary spheres and, under conducive conditions, differentiate into various cell types [36, 37]. These cells exhibit not only in vitro trilineage differentiation but also contribute to diverse in vivo outcomes, including the development of bone-like structures, endothelial cells, cardiomyocytes, hepatocytes, and pancreatic cells. VSELs demonstrate migratory capabilities, responding to specific tissues during migration assays and showing a prompt response to tissue damage. Moreover, they exhibit stress resistance, surviving high doses of gamma-irradiation and extrinsic heat stress, showcasing their robust nature in adverse conditions (Table 1, Fig. 1).

Comprehensive analyses have been conducted on the proteome and transcriptome of VSELs, revealing dynamic profiles that contribute to their functional diversity. Researchers have diligently investigated the molecular characteristics of VSELs to understand their developmental origin. By studying highly purified double-sorted VSELs from murine bone marrow under steady-state conditions, it has been observed that these cells express genes associated with both epiblast specification (e.g., Stella, Prdm14, Fragilis, Blimp1, Nanos3, and Dnd1) and primordial germ cells (PGCs). Notably, PGC-specific genes like Dppa2, Dppa4, and Mvh, which are characteristic of late-migratory PGCs, were also expressed. Authors hypothesized that VSELs maintain quiescence in adult tissues through mechanisms similar to those governing PGC quiescence, involving epigenetic modifications of paternally imprinted genes [38]. These findings suggest that VSELs modify the imprinting of early-development imprinted gene loci (e.g., Igf2–H19), rendering them resistant to insulin-like growth factor signaling. These insights strengthen the link between PGCs and VSELs as potential precursors for long-term tissue regeneration.

Some studies suggest that VSELs may possess immunomodulatory capabilities, indicating their potential interactions with the immune system. Claims have been made that VSELs could regulate the immune response, potentially enhancing it in cases of infections or injuries and attenuating it in conditions of autoimmune hyperactivity. However, these findings have been reported primarily on the websites of private regenerative medicine centers, lacking validation through scientific evidence.

On the other hand, several clinical trials suggest that VSELs might exhibit immune-privileged properties, potentially enabling transplantation across histocompatibility barriers without triggering an immune response. Nevertheless, further research is necessary to comprehensively understand the underlying mechanisms and to develop effective therapies utilizing VSELs [39].

VSELs are currently being investigated in clinical trials for various applications [40, 41]. In the context of knee osteoarthritis, researchers are exploring the safety and efficacy of autologous VSELs by injecting them into the affected knee. Additionally, VSELs are being evaluated for facial skin antiaging, where they are injected into specific areas. Furthermore, these stem cells are being studied for their potential role in addressing organic erectile dysfunction. Overall, VSELs hold promise as a fascinating avenue for tissue regeneration and aging-related therapies.

A significant application of VSEL cells has been explored in diabetes repair. VSELs, isolated from mouse bone marrow, were analyzed for specific markers and demonstrated the ability to differentiate into various cell types. Upon intravenous injection into mice with pancreas damage, VSELs migrated to the pancreas, survived, and resulted in a significant decrease in blood glucose levels for at least two months. The mice also experienced gradual weight gain [42]. This groundbreaking research suggests that VSELs could be a promising strategy for treating diabetes and other regenerative diseases, offering a viable alternative to traditional stem cell therapies [35, 43].

In summary, VSELs present a unique combination of migratory, stress-resistant, and differentiation features, positioning them as resilient contributors to tissue regeneration and repair. This makes them promising candidates for therapeutic applications in regenerative medicine, akin to MUSE cells, showcasing distinct characteristics in diverse biological contexts.

### Marrow isolated adult multilineage inducible (MIAMI) cells

Marrow Isolated Adult Multilineage Inducible (MIAMI) cells were discovered in 2004 by Gianluca D'Ippolito and his team [47]. These cells have sparked significant interest in the field of regenerative medicine due to their distinctive characteristics and promising therapeutic potential. The process of isolating MIAMI cells involves a specialized expansion and selection procedure that emulates the in vivo microenvironment of primitive stem cells in the bone marrow. This process entails co-culturing adherent and non-adherent marrow cells on fibronectin under low oxygen conditions (3%) [48]. Compared to human mesenchymal stem cells, which exhibit a typical fibroblastic morphology with few long and thin cellular processes, MIAMI cells appear smaller, with a more compact and rounded cytoplasm. The remarkable ability of MIAMI cells to differentiate into various lineages—mesodermal, ectodermal, and endodermal—makes them versatile for addressing diverse tissue regeneration needs. Surface marker analysis plays a key role in identifying and characterizing these cells. They express specific markers such as CD29, CD63, CD81, CD122, CD164, cMet, BMPR1B, and NTRK3, while significantly lacking expression of markers like CD34, CD36, CD45, cKit, and HLA-DR. This distinctive marker profile contributes to their unique identity. Additionally, MIAMI cells express typical embryonic stem cell markers such as Sox2, Nanog, Oct-4, and Rex-1, along with strong telomerase expression, indicating their pluripotent characteristics, despite exhibiting a rounded and compact morphology with a high nucleus-to-cytoplasm ratio [47]. MIAMI cells isolated from various donors' bone marrow show consistently similar genetic expression, regardless of age and sex, and appear to share more proteins with human embryonic stem cells than with mesenchymal stem cells, but without the potential to form teratomas, as they have been demonstrated to be non-cancerogenic [48]. Additionally, the expression level of distinctive markers of MIAMI cells remains constant regardless of age and gender. Furthermore, although the proportion of MIAMI cells compared to the total marrow nucleated cells decreases from 0.01% at the age of 3 to 0.0018% at the age of 45, their overall number remains stable after the age of 45.

Due to these characteristics, MIAMI cells are currently under investigation in various clinical applications, including tissue regeneration [49]. MIAMI cells express numerous markers similar to embryonic stem cells, they do not possess complete self-renewal capacity; however, they appear to respond to specific molecular signals in the right environmental conditions to induce self-renewal. This can be exploited to manipulate these cells to become more stable, maintain their pluripotency, and support their immunoregulatory properties for longer periods. Importantly, MIAMI cells show rapid proliferation without signs of senescence, ensuring the maintenance of their differentiation potential during prolonged culture periods, which is crucial for scalability and potential clinical applications. MIAMI cells demonstrate migratory capabilities to damaged tissues and immunomodulatory capacity, suggesting their potential in tissue repair and immunomodulation [50, 51]. In therapeutic applications, MIAMI cells have shown promise in various pathologies, such as in the treatment of peripheral vascular disease (PVD). Studies using a murine model of critical limb ischemia have demonstrated that the combination of MIAMI cells with a bilayer electrospun gelatin B nanofiber construct (BIC) significantly improved limb recovery compared to single treatments [52]. This combined approach led to improved blood flow restoration, reduced ischemia and necrosis, and prevention of intermuscular adipose tissue infiltration (IMAT). However, further research and clinical studies are essential to unveil the full therapeutic potential of MIAMI cells and establish their role in treating a variety of diseases and conditions.

### Multipotent adult progenitor cells (MAPCs)

The multipotent adult progenitor cells (MAPCs) were first identified in human bone marrow and subsequently confirmed in animal models, such as mice and rats. MAPCs have demonstrated a remarkable ability to differentiate into a variety of cell lineages, including mesodermal, ectodermal, and endodermal lineages. These cells can be isolated from various tissue sources, including bone marrow, brain, muscle and bone tissue [53, 54]. However, isolating MAPCs from bone marrow has been one of the most common and widely studied methods to date. The process of isolating MAPCs from bone marrow involves several key steps that have been developed and optimized over the years. One distinctive feature of this process is the use of low-oxygen conditions, typically around 5%, during cell isolation. This hypoxic environment mimics the physiological conditions present in the bone marrow and promotes the maintenance of the unique properties of MAPCs. Once a critical mass of cells is reached in culture, MAPCs can then be selected using specific cell surface markers through flow cytometry techniques, allowing for the separation of MAPCs based on their expression of specific markers such as CD44, CD13, CD73, CD90, CD105, CD31, and CD49d [53]. Despite MAPCs may be present in a population of MSCs, the crucial points that define MAPCs compared to MSCs essentially lie in their different origins, not only mesodermal, and surface marker expressions. These differences define distinct potentials, such as a broader differentiative capacity, a more pronounced immunomodulatory capacity, and better performance in cell therapy. One of the most remarkable features of MAPCs is their ability to surpass the differentiation potential of traditional MSCs, which are also frequently used in the field of regenerative medicine. Therefore, not only do they possess the ability to differentiate into a variety of cell types, like MSCs, but they also exhibit exceptional plasticity and adaptability, allowing them to cross lineage barriers more completely and efficiently. MAPCs demonstrate immunomodulatory properties that go beyond those of MSCs. They can modulate immune responses, playing a role in regulating inflammation and promoting a favorable environment for tissue healing. This immunomodulatory behavior of MAPCs makes them particularly interesting for application as universal donors, as they can be transplanted into patients without the risk of immunological rejection. The option to use MAPCs as universal donors is highly appealing in regenerative medicine, as it reduces the need to find a matching donor and the risk of tissue compatibility complications. The clinical applications of multipotent adult progenitor cells (MAPCs) are extremely broad and promising, with various pieces of evidence confirming their efficacy in crucial therapeutic contexts. One of the most interesting aspects is the use of MAPC secretome, known as MAPC-conditioned medium (MAPC-CM), as a therapeutic agent for wound healing. This secretome contains a rich mixture of growth factors, cytokines, and other bioactive molecules that influence a series of key processes in tissue repair. Studies conducted on animal models with excisional wounds have shown that the application of MAPC-CM can promote cell migration, stimulate cell proliferation, promote collagen deposition, and enhance the formation of new blood vessels, known as angiogenesis. These combined effects contribute to the rapid and effective healing of damaged tissues. Furthermore, clinical studies have demonstrated that MAPCs can have a significant impact on reducing myocardial scars in patients who have suffered from a myocardial infarction [55]. This is particularly relevant considering that myocardial scars can compromise long-term cardiac function and increase the risk of cardiovascular complications. MAPCs, due to their ability to differentiate into cardiac cells and their modulating effect on the surrounding microenvironment, can contribute to the regeneration of damaged cardiac tissue and the reduction of scars, thereby improving cardiac function and reducing the risk of complications. In the context of stroke recovery, the MASTERS study has highlighted that MAPCs, particularly the Multistem type, can offer significant benefits if administered early within the first 36 h after the stroke [55]. This underscores the crucial importance of optimized timing in stem cell therapies. MAPCs can act by reducing inflammation, promoting the regeneration of damaged brain tissues, and improving neurological function, thereby contributing to the recovery process after a stroke. Transcriptomic analyses have also been performed, providing important insights into the differences in differentiation potential between MAPCs and traditional MSCs [56]. These analyses have revealed that MAPCs show a greater inclination towards endothelial differentiation, namely the formation of cells that comprise blood and lymphatic vessels. This characteristic has been supported by in vitro experiments, such as Matrigel plug tests, which simulate the formation of blood vessels in a three-dimensional environment. MAPCs thus appear to express genes that are involved in angiogenesis, the process through which new blood vessels are formed from pre-existing ones, promoting tissue growth and repair [57, 58]. On the other hand, MSCs seem to show a greater propensity towards differentiation into cartilage (chondrogenic) and bone (osteogenic) tissue cells. In summary, transcriptomic analyses have highlighted that MAPCs and MSCs present significant differences in their differentiation potential, with the former showing a greater inclination towards blood vessel formation and the latter towards the formation of cartilage and bone tissues. These differences can have crucial implications in the context of regenerative medicine, allowing for the targeted use of each cell type based on the specific needs of the patient and the pathological condition to be treated. In conclusion, MAPCs exhibit exceptional characteristics that make them valuable in regenerative medicine. Their ability to differentiate into a wide range of cell lineages, together with their immunomodulatory properties and distinct transcriptomic profiles, makes them versatile players in the treatment of a variety of pathologies. Research efforts continue to fully explore and exploit the therapeutic potential of MAPCs, with the aim of improving the health and quality of life of patients suffering from chronic diseases and severe injuries.

### Small blood stem cells (SBSCs)

Identified in human peripheral blood, Small Blood Stem Cells (SBSCs), as reported by Filidou et al. [44], exhibit specific characteristics and differentiation potential, solidifying their significance in the landscape of stem cell biology. SBSCs showed bulk in vitro trilineage differentiation as demonstrated by RT-qPCR and ICC, while specific details about clonal in vitro trilineage differentiation and bulk in vivo differentiation are not provided (Table 1, Fig. 1).

The population of SBSCs is distinguished by its unique expression profile, encompassing pluripotent embryonic markers, hematopoietic markers, and mesenchymal markers. This heterogeneity suggests a multifaceted nature of SBSCs, contributing to their potential in various biological processes.

Notable markers and factors associated with SBSCs encompass pluripotent and embryonic markers, exemplified by the expression of NANOG, SSEA-3, SSEA-4 and CXCR4 highlighting their potential for multilineage differentiation. SBSCs exhibit a distinctive co-expression of hematopoietic markers (CD45) and mesenchymal markers (CD90, CD29, CD105, PTH1R), suggesting their association with both blood cell development and mesenchymal lineage differentiation (Table 1). Quantitative proteomic profiling of SBSCs has identified diverse stem cell markers, including CD9, ITGA6, MAPK1, MTHFD1, STAT3, HSPB1, and HSPA4, enriching the understanding of their molecular composition (Fig. 1). Moreover, SBSCs harbor transcriptional regulatory complex factors like STAT5B, PDLIM1, ANXA2, ATF6, and CAMK1, contributing to their regulatory capabilities. This comprehensive expression profile underscores the heterogeneity and versatility of SBSCs, positioning them as a unique subset of stem cells with the potential to play a pivotal role in diverse biological processes. The isolation of SBSCs involves a protocol with serial centrifugation, facilitating their separation and collection from peripheral blood [45]. Specific details about the immunomodulatory capacity of SBSCs are not provided, highlighting avenues for further exploration to comprehensively understand their therapeutic potential in regenerative medicine. This characterization emphasizes the importance of various attributes for the evaluation and potential application of these stem cell populations.

Anyway, recent findings investigate the safety and tolerability of SBSC in dental implantation for patients with severe bone defects. Nine patients received different doses of SB cells, and evaluations were conducted through computed tomography (CT) scans and comprehensive chemistry panel testing. The trial, spanning six months, revealed no severe adverse effects, with observed improvements in bone mineral density (BMD) and stress levels. Elevations in specific cytokines and chemokines indicated SBSC-triggered responses for local tissue repair [46]. The findings support the well-tolerated use of SB cells in dental implantation, suggesting their potential for accelerating osseointegration in high-risk patients.

Coordinating embryonic, hematopoietic, and mesenchymal markers, along with the presence of various stem cell-related factors, accentuates the intriguing nature of SBSCs, warranting further investigation in the realm of stem cell research.

## Markers and methodologies used for isolation

Analyzing the surface markers of the five considered stem cell populations and isolation methods, SSEA-3 emerges as a key marker extensively used in the isolation process. SSEA-3 plays a pivotal role in identifying and purifying these stem cell populations [16].

The chemokine receptor CXCR4 has been identified as a relevant marker associated with VSELs. CXCR4, also known as C-X-C chemokine receptor type 4, plays a role in the migration and homing of stem cells. CXCR4 is expressed on the surface of VSELs, and its interaction with its ligand, SDF-1 (stromal cell-derived factor 1), is considered crucial for the mobilization and homing of VSELs in various tissues [32]. This interaction is implicated in the trafficking of VSELs to areas of tissue damage or injury, where they may contribute to regenerative processes. The presence of CXCR4 on VSELs is a notable characteristic and contributes to the understanding of the homing and migration mechanisms that these stem cells employ.

CD133, also known as prominin-1, is a surface marker associated with VSELs. CD133 is a glycoprotein and is often utilized as one of the distinctive markers for isolating and characterizing VSELs.

In addition to SSEA-3 and CXCR, several other markers have been mentioned for isolating specific populations of stem cells. For instance, CD45 and CD90 have been identified as co-expressed markers in some SBSCs, emphasizing their association with blood cell development. CD29, CD105, and PTH1R have been recognized as mesenchymal markers expressed by SBSCs, indicating their ability to differentiate into mesenchymal lineages.

For MIAMI cells, CD122, CD29, CD63, CD81, CD164, CD90, and SSEA-4 have been cited as surface markers. These markers provide a distinctive profile for the identification and characterization of MIAMI cells. In the context of MAPCs, markers such as CD44, CD13, CD73, CD90, CD105, CD31, CD49d have been indicated as distinguishing elements.

Notably, methods such as Fluorescence-Activated Cell Sorting (FACS) and Magnetic-Activated Cell Sorting (MACS) have been employed for their precision in isolating cells expressing specific markers, like SSEA-3 [17]. These techniques enable the attainment of a more homogenous cell population, concentrating solely on those cells expressing the targeted marker. In contrast, the isolation method involving bone marrow under low oxygen tension (3% O2) emphasizes a distinct approach, suggesting the importance of the microenvironment in which stem cells reside.

FACS and MACS are particularly advantageous in achieving a higher degree of purity in isolated populations, ensuring that the isolated cells predominantly express the desired surface markers. This precision is crucial for subsequent analyses and applications, enhancing the reliability of research outcomes. On the other hand, methods like isolation from bone marrow under low oxygen tension might yield more heterogeneous populations, potentially capturing a broader range of stem cells with diverse characteristics.

The choice of isolation method significantly influences the purity and homogeneity of the obtained stem cell populations. While FACS and MACS offer a more defined and targeted approach, other methods might capture a broader spectrum of stem cell phenotypes.

## Role of cell cycle phases in the isolation of pluripotent stem cells

The dynamic nature of stem cells, as they undergo cycling, implies a constantly changing phenotype. This characteristic serves as a protective mechanism, preventing catastrophic toxicity by allowing stem cells to exhibit different phenotypes at relatively short intervals during the cell cycle. The concept of a "stem cell calculus" is proposed, wherein changes in phenotype throughout the cell cycle represent individual components, and the overall outcome is an integration of these changes. Various studies on different stem cell types align with this model. Notably, research on highly purified LRH stem cells, even when isolated at different cell cycle points, has revealed significant heterogeneity. This observed heterogeneity, while present at the cellular level, still demonstrates overall patterns of differentiation. An analogy is drawn to the decay of radioactive substances, where individual atomic behavior appears random, but when observed as a whole, it follows a predictable pattern. This suggests that the regulation of the stem cell population as a whole, rather than individual cells, involves control mechanisms influencing birth and death probabilities [8, 59–61].

This perspective raises questions about the previously-dismissed significance of the stem cell assay CFU-s (Colony-forming Unit Spleen), as it did not correlate with studies on purified stem cells. Given the observed heterogeneity in purified stem cells, the importance of CFU-s as a stem cell assay may need reconsideration. Recent emphasis on single-cell RNA analysis has revealed heterogeneity in different cell populations, including murine hematopoietic stem cells. Even in highly purified cells, small cell cycle progressions likely contribute to observed heterogeneity [62].

The proposed model suggests the existence of a universal stem cell encompassing hematopoietic LT-HSC (long-term hematopoietic stem cells) and various non-hematopoietic stem and progenitor cells, forming a continuum related to the cell cycle [8]. The differentiation of this stem cell relies on cell cycle-related changes in differentiation potential, illustrated by marker expressions like B220 and Gr-1, along with data on megakaryocyte development. The stem cell's tissue residence, modulated by extracellular vesicles, plays a crucial role, allowing transformation into non-hematopoietic tissue-specific stem cells. This model accounts for constant heterogeneity in different stem and progenitor cell classes, attributing it to continual phenotypic changes as the stem cell progresses through the cell cycle. The model is conceptualized as a stem cell calculus, where cycle-related phenotype changes represent derivatives, and the overall population outcome is the integral. While previous studies have provided valuable insights into purified LT-HSCs at specific cell cycle phases, future progress in the field involves characterizing the entire stem cell population.

This may lead to stem cell misclassification and identification, see the discussion.

## Discussion

The analysis of the five stem cell populations highlights several common features that are of particular interest in the context of regenerative medicine. Firstly, Multilineage-differentiating Stress Enduring (MUSE) cells, Very Small Embryonic-Like (VSEL) Cells, Small Blood Stem Cells (SBSC), Marrow Isolated Adult Multilineage Inducible (MIAMI) Cells, and Multipotent Adult Progenitor Cells (MAPC) share the presence of pluripotent embryonic markers and the ability to differentiate into a wide range of cell types, spanning the three germ layers. The notable similarity in differentiative potential suggests a possible common origin, even if contrasting data are present in literature on this subject.

MUSE cells are the most extensively studied cell types among the five above reported stem cells and hold promise for regenerative medicine. These well-characterized cells exhibit extraordinary potential for repairing damaged tissues. Similarly, VSELs have also been the focus of intense research and possess equally intriguing characteristics. Both cell types share several key features: MUSE cells and VSELs express the SSEA-3 antigen [63, 64], which plays a role in cellular functions and differentiation; both cell types can migrate to damaged tissues, contributing to tissue repair; these cells demonstrate remarkable resilience to environmental stressors and adverse conditions. However, there are some notable differences between them. MUSE cells are mainly derived from mesenchymal tissues, while VSELs are believed to be derived from primordial germ cells or other embryonic precursors [65]. VSELs are exceptionally small, with diameters ranging from 3 to 5 µm, whereas MUSE cells are reported to be larger.

The extremely small size reported for VSEL, ranging from 3 to 5 µm, raises questions and triggers skepticism within the scientific community [37]. Cells of such diminutive dimensions are seldom encountered, and the presence of cells this small within the context of VSELs has been a subject of debate. The rarity of cells with such minuscule sizes in the cellular landscape introduces a level of skepticism regarding their actual existence and biological potential. Concerns have been voiced within the scientific community regarding the possibility that measurements of VSEL sizes may be influenced by various analytical techniques, emphasizing the need for further characterization of phenotype and isolation methodologies to more conclusively establish the true nature and size of these particular cells.

Despite these differences, both MUSE and VSEL cells offer exciting avenues for regenerative therapies, and ongoing research aims to harness their potential for treating various diseases and injuries.

In a recent paper by Oguma et al. [66], a comprehensive analysis of the transcriptome of MUSE cells at the single-cell level was conducted, drawing a comparative assessment with the transcriptome of MSCs. The study focused also on evaluating the expression profiles of various markers associated with VSELs within both MUSE cells and MSCs. VSELs, as defined by Shin et al. (2012), are characterized by their positivity for CXCR4, along with the expression of epiblast-related markers (GBX2, FGF5, NODAL) and primordial germ cell-related markers (DPPA3 [Stella], PRDM1 [Blimp1], PRDM14), while being negative for PTPRC (CD45). Intriguingly, neither MUSE nor MSCs expressed these specific marker genes, with the exception of FGF5, which exhibited higher expression levels in MSCs compared to MUSE [66].

VSELs also express additional markers, such as SSEA-4, and share similarities with germ cells, expressing markers like DDX4/VASA and PRDM14. In vitro, these VSELs remain quiescent, except in ascites, and become highly activated after exposure to valproic acid and follicle-stimulating hormone (FSH). VSELs spontaneously form aggregates resembling tumor-like structures or grow into larger cells resembling oocytes. Several studies propose a germinal origin for VSELs, while MUSE cells are found in both stromal tissues like bone marrow and tissues from the umbilical cord. While certain conditions may lead to uncontrolled proliferation of VSELs, resulting in tumor formation, numerous studies have demonstrated the non-tumorigenic nature of MUSE cells [26, 33]. This stark contrast in tumorigenicity further underscores the distinctive characteristics between VSELs and MUSE cells, emphasizing the importance of comprehending their unique molecular profiles and biological behaviors for their potential applications in regenerative medicine.

This differential expression pattern provides valuable insights into the distinctive molecular profiles of MUSE cells and VSEL, emphasizing the need for a nuanced understanding of their biological characteristics and potential applications in regenerative medicine.

SBSC exhibit considerable overlap in characteristics with VSELs, except for the expression of CD45. This commonality suggests a shared profile related to pluripotency and regenerative potential. Furthermore, a comparative analysis with MUSE cells reveals intriguing parallels, as both SBSC and MUSE cells express SSEA-3. Interestingly, SBSC isolated from peripheral blood also exhibit CD45 expression, aligning them with MUSE cells in this aspect. However, a notable distinction remains in terms of cell size. These findings contribute to a nuanced understanding of the unique features and potential applications of SB cells, positioning them within the broader landscape of stem cell populations and highlighting both shared and distinct attributes.

MAPCs have attracted attention in biomedical research due to their remarkable capacity to differentiate into various cell types derived from the three embryonic germ layers: endoderm, mesoderm, and ectoderm. MUSE, VSEL, and MAPC stem cells can be isolated from stromal tissues, but MAPC cells uniquely possess the ability to be isolated from several organs. Recent studies indicate the potential presence of MAPCs in other adult tissues, such as the liver and brain, expanding their possible sources of isolation. Molecular distinctions between MAPC and other stem cells, including the differential expression of pluripotent and surface markers, may reflect the diverse origins and differentiation potentials of these cell populations. Both MUSE and MAPCs have demonstrated the ability to differentiate into a wide range of cell types; however, their effectiveness or differentiation potential may vary in specific experimental contexts or in relation to the preferred cell types. For instance, MAPCs might exhibit a greater predisposition to differentiate into specific cell types compared to MUSE cells, or vice versa, depending on experimental conditions or the cellular environment [58]. Concerning tumorigenicity, MUSE cells have been consistently described as non-tumorigenic in various studies, making them promising for clinical applications without the risk of tumor formation. In contrast, MAPCs may show tumorigenic potential in certain contexts or if not adequately controlled during culture, raising concerns about their therapeutic use.

MAPCs have emerged as a subject of controversy within the scientific community, primarily due to challenges associated with their reproducibility. The reliability and consistency of research findings related to MAPCs have been called into question, leading to the retraction of several studies that initially reported on these cells. The inherent difficulty in replicating experimental outcomes with MAPCs has raised concerns about the robustness and validity of the scientific evidence surrounding their properties and potential applications. These uncertainties underscore the importance of rigorous experimental design, standardization of methodologies, and comprehensive validation processes to address the reproducibility issues associated with MAPCs and establish a more reliable foundation for their characterization and therapeutic exploration. In summary, although MAPCs share some characteristics with the other population of stem cells, such as the ability to differentiate into different cell types, there are significant differences in their tissue origins, surface markers, tumorigenic potential, and other biological characteristics, which make them distinctive and potentially useful for different applications in regenerative medicine.

MIAMI cells are adult cells with interesting therapeutic potential but distinctive characteristics. Indeed, the isolation of MIAMI cells, to date, occurs solely from the bone marrow aspirates, while MUSE cells and VSEL can be isolated from a variety of stromal tissues. Nonetheless, these cells have sparked significant interest in the field of regenerative medicine due to their versatility and potential in treating bone lesions and musculoskeletal disorders. Unlike MUSE stem cells, known for their remarkable resistance to stress, MIAMI are not generally considered stress-resistant. This may impact their utility in clinical contexts where stress conditions are a significant factor. However, MIAMI cells have demonstrated to promote blood vessel formation and reduce inflammation and necrosis in ischemic tissues. This is attributable to the secreted factors they release into the surrounding environment, as identified by secretome analysis. Regarding surface markers, both for MIAMI and MAPC cells, the expression of SSEA-3 still needs to be evaluated.

In-depth studies on the proteome and transcriptome of these cell subpopulations could be pivotal to better understand the molecular basis of their unique characteristics and potential common origins. Identifying key expressed genes and proteins could shed light on the regulation of molecular pathways involved in pluripotency and cell differentiation.

The cell cycle plays a predominant role in influencing the differentiation capabilities of stem cells and the expression of various stem cell surface markers. In the context of the proposed universal stem cell hypothesis, a comprehensive investigation into the cell cycle phases of pluripotent stem cells isolated from stromal tissues becomes crucial. It is conceivable that the expression of specific markers in pluripotent stem cells might vary across different cell cycle phases. For instance, certain markers may be expressed during the G1 phase but not in other phases like S phase. Analyzing marker expression at distinct cell cycle stages could provide valuable insights into the regulatory mechanisms governing pluripotent stem cells. Correlating these cell cycle phases with the differentiation capacities across the three embryonic germ layers further enhances our understanding. This detailed analysis is instrumental in uncovering both similarities and differences within these stem cell populations. Ultimately, exploring the expression dynamics of stem cell markers throughout the cell cycle holds great promise for unraveling the intricacies of pluripotent stem cell behavior and refining our comprehension of their unique characteristics.

However, it is worth noting that the high variability in results across different laboratories could be attributed to technical differences in the isolation and culture procedures of these stem cells. Standardizing work methodologies could help reduce this variability and provide more consistent results, thereby facilitating a more accurate understanding of each population's intrinsic characteristics.

**Fig. 2:**
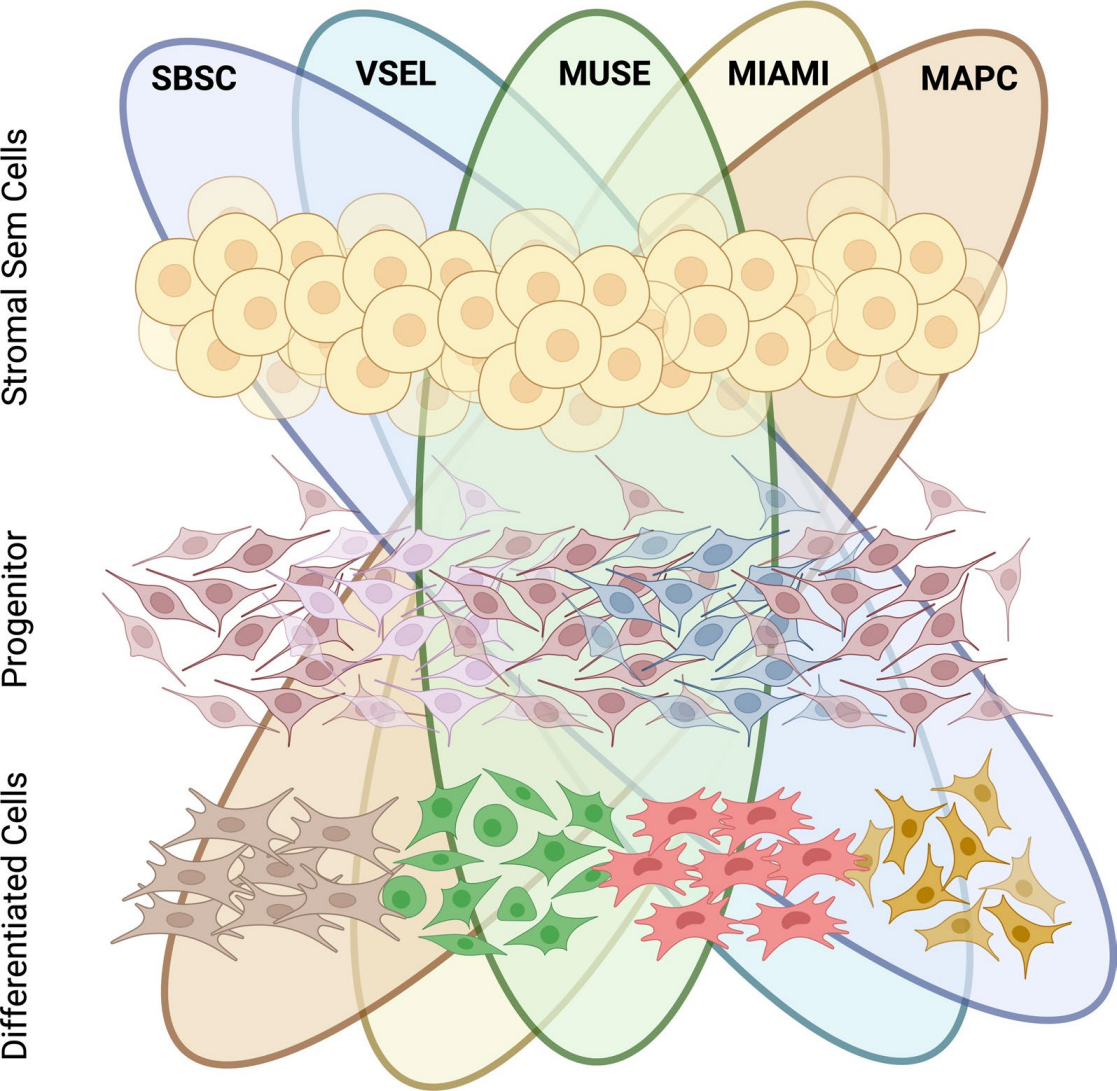
Correlation Hypothesis Among Different Stromal/Stem Cell Lines: within stromal tissues and peripheral blood, various populations of multipotent and pluripotent stem cells (including MUSE cells, VSEL, MAPC, MIAMI, SBSC) exist. These populations share numerous common characteristics, suggesting potential interrelations or overlaps. Factors such as different isolation protocols, distinct cell cycle stages, or diverse niches could contribute to the isolation of these subpopulations. Each selected subpopulation (MUSE, VSEL, MAPC, MIAMI, SBSC) possesses specific progenitors and differentiation potential. Given the overlapping nature of stem cell populations, it is plausible that these features exhibit commonalities. However, further investigation is needed to determine whether stromal tissues harbor distinct subsets of stem cells with overlapping features or contains a single stem population, with subtypes, which can be isolated based on the aforementioned factors

Taking into consideration the above-reported findings, the differences in biological properties among the various stromal stem cell populations so far described may be due to the fact that they are truly distinct cell populations (Fig. 2). Alternatively, it must be remembered that stem cells are inherently heterogeneous. This implies that the stem cell niche hosts different subpopulations of stem cells, each presenting subtle differences in stemness and lineage potential [67]. The isolation and cultivation procedures of the aforementioned stromal stem cells may have selected a specific stem cell subpopulation from the larger population of stromal stem cells.

This scenario is further complicated by the fact that every single cell population may exhibit differences according to the cell cycle stage of its components. Finally, confounding issues may also arise from differences in the evaluation of biological properties, either in vitro or in vivo*.*

## Conclusions and future perspectives

In conclusion, multipotent/pluripotent adult stem cell (PSC) populations represent an extraordinary resource for regenerative medicine, offering therapeutic possibilities for a broad spectrum of pathologies. However, the confusion arising from the diversity of PSC types and their unique characteristics emphasizes the need to isolate and characterize these populations in common laboratories. Only through detailed analyses of the proteome, secretome, and transcriptome can we clarify the overlaps and differences between these stem cells, contributing to a deeper understanding of their origins and therapeutic potential.

The future of PSC research should focus on standardizing methodologies and conducting in-depth analyses in shared laboratories. This approach could not only reduce variability in results but also facilitate scientific collaboration to maximize the therapeutic potential of pluripotent stem cells. Furthermore, ongoing research into optimizing the timing of therapies underscores the need for further investigations to refine treatment strategies across different clinical conditions.

## Abbreviations

ASCs: Adipose tissue-derived stem/stromal cells
FACS: Fluorescence-activated cell sorting
HSC: Hematopoietic stem cells
MACS: Magnetic-activated cell sorting
MAPC: Multipotent adult progenitor cells
MIAMI: Marrow-isolated adult multilineage inducible cells
MSCs: Mesenchymal stem/stromal cells
MUSE: Multilineage differentiating stress enduring cells
PGCs: Primordial germ cells
PSC: Pluripotent stem cells
S1P: Sphingosine-1-phosphate
SBSC: Small blood stem cells
VSELs: Very small embryonic-like stem cells
